# Anti-*Toxoplasma gondii* Effects of a Novel Spider Peptide XYP1 In Vitro and In Vivo

**DOI:** 10.3390/biomedicines9080934

**Published:** 2021-08-01

**Authors:** Yuan Liu, Yaqin Tang, Xing Tang, Mengqi Wu, Shengjie Hou, Xiaohua Liu, Jing Li, Meichun Deng, Shuaiqin Huang, Liping Jiang

**Affiliations:** 1 Department of Parasitology, Xiangya School of Medicine, Central South University, Changsha 410013, China; 186511077@csu.edu.cn (Y.L.); hanyin998@csu.edu.cn (Y.T.); wumengqi@csu.edu.cn (M.W.); HSJ1199@csu.edu.cn (S.H.); liuxiaohua0306@csu.edu.cn (X.L.); lijing0807@csu.edu.cn (J.L.); huangshuaiqin@xmu.edu.cn (S.H.); 2 Hunan Key Laboratory for Conservation and Utilization of Biological Resources in the Nanyue Mountainous Region, Hengyang Normal University, Hengyang 421008, China; xtang2011@sina.com; 3 Department of Biochemistry and Molecular Biology, School of Life Sciences, Central South University, Changsha 410013, China; dengmch@csu.edu.cn; 4 China-Africa Research Center of Infectious Diseases, Xiangya School of Medicine, Central South University, Changsha 410013, China

**Keywords:** *Toxoplasma gondii*, spider peptide XYP1, anti-parasitic activity, functional mechanism, drug discovery

## Abstract

Toxoplasmosis, caused by an obligate intracellular parasite *Toxoplasma gondii*, is one of the most prevalent zoonoses worldwide. Treatments for this disease by traditional drugs have shown numerous side effects, thus effective alternative anti-*Toxoplasma* strategies or drugs are urgently needed. In this study, a novel spider peptide, XYP1, was identified from the cDNA library of the venom gland of the spider *Lycosa coelestis*. Our results showed that XYP1 has potent anti-*Toxoplasma* activity in vitro and in vivo. Specifically, treatment with XYP1 significantly inhibited the viability, invasion and proliferation of tachyzoites with low cytotoxicity (IC_50_ = 38.79 μΜ) on human host cells, and increased the survival rate of mice acutely infected with *T. gondii*. Next, scanning electron microscopy, transmission electron microscopy and RNA sequencing were employed to further explore the functional mechanism of XYP1, and the results indicated that XYP1 causes membrane perforation, swelling and disruption of tachyzoites, which could be closely associated with differential expression of several membrane-associated proteins including HSP29. In conclusion, XYP1 may be a promising new drug candidate for the treatment of toxoplasmosis.

## 1. Introduction

*Toxoplasma gondii* (*T*. *gondii*), an obligate intracellular parasite, can infect humans and various warm-blooded animals and cause toxoplasmosis [1]. *T*. *gondii* can be transmitted to the fetus vertically through the placenta of pregnant women with toxoplasmosis, causing spontaneous abortion and stillbirth [2]. Toxoplasmosis is the most common opportunistic disease in human immunodeficiency virus (HIV) patients, which is fatal to immunocompromised individuals [3].

For the prevention and treatment of toxoplasmosis, there are many difficulties and bottlenecks in the development of anti-toxoplasmosis vaccines and drugs. Up to now, there is still no approved vaccine for human toxoplasmosis, and for the control of toxoplasmosis mainly rely on sulfadiazine (SFZ), pyrimethamine and acetylspiramycin [4,5]. However, these traditional drugs have several problems including high toxicity, side effects, drug resistance, etc. [6,7]. Therefore, the development of new alternative therapeutic options or drugs against toxoplasmosis is urgently needed.

At present, research on anti-*Toxoplasma* drugs mainly includes: anti-*Toxoplasma* effects of clinical drugs (pravastatin and simvastatin); compounds extracted from natural products of animals and plants; and artificial small molecular compounds [8,9,10,11]. In recent years, owing to the simple structure, high targeting ability and no or low drug resistance, linear peptides have drawn increasing attention from many researchers working on the development of new anti-parasitic drugs [6,7]. Spider venom is a huge potential drug repository which has not been widely exploited. Our previous studies suggested that two spider venoms derived from *Ornitoctonus huwena* and *Chilobrachys jingzhao* exhibited potent effects on *T. gondii* in vitro and in vivo [12]. It was also reported that the *Haemaphysalis longicornis* longicin P4 peptide can cause *Toxoplasma* tachyzoite destruction [13]. Lycosin-I, a 24-residue cationic antimicrobial peptide derived from the venom of the spider *Lycosa singoriensis* (*L*. *singoriensis*) showed inhibitory effects on *T. gondii* and protective effects on the host [14]. This research indicated that the spider peptides could be effective drug candidates against toxoplasmosis. *Lycosa coelestis* (*L. coelestis*), distributed in mountainous areas of southern China, is the natural enemy of many common pests. Interestingly, *L. coelestis* preys on pests with its venom, but could not be affected by various pathogens from the complex environment, indicating that the venom may contain some effective anti-pathogen agents. No studies on antibacterial or antiparasitic peptides using the spider *L. coelestis* have been carried out.

Considering the side effects and drug resistance of previous anti-*T*. *gondii* drugs and compounds, new therapeutic strategies for toxoplasmosis are urgently needed. To find a safer and more effective drug or prodrug candidate, in the present study, the complementary DNA (cDNA) library of the venom gland of spider *L. coelestis* was established and a novel spider peptide XYP1 was identified and characterized. Experimental models infected with *T. gondii* in vitro and in vivo were used to evaluate the anti-*T. gondii* effects of XYP1. Besides, to explore the molecular mechanism involved, RNA sequencing (RNA-seq) and quantitative real-time PCR (qRT-PCR) were employed, which has not been previously reported.

## 2. Materials and Methods

### 2.1. Animals

Specific-pathogen-free (SPF) eight-week-old female Kunming (KM) and BALB/c mice were purchased from the Department of Laboratory Animals, Central South University (Changsha, China). The study was conducted according to the guidelines of the International Conference on Harmonization, and approved by the Ethics Committee of School of Basic Medical Science, Central South University, Changsha, China (protocol code 2020KT-11 and date of approval 7 April 2020).

### 2.2. Cell Culture and Maintenance of T. gondii

Human foreskin fibroblasts (HFFs) were cultured in Dulbecco’s modified Eagle’s medium (DMEM, Gibco Co., Gaithersburg, MD, USA) supplemented with 10% fetal bovine serum (FBS; Gibco Co., USA) and 1% antibiotics (10,000 U/mL penicillin and 10 mg/mL streptomycin solution) (HyClone Co., Logan, UT, USA) and maintained in an incubator containing 5% CO_2_ at 37 °C.

*T. gondii* tachyzoites was primarily maintained in KM mice by serial intraperitoneal passage [15]. Tachyzoites harvested were intraperitoneally injected to the next KM mouse or seeded to infect HFFs.

### 2.3. Peptide Synthesis, Agents and Solutions 

The peptide XYP1 was synthesized by the Pepmic Company, Suzhou, China and its molecular mass and purity were determined by Ultraflex™ TOF/TOF MS spectrometer. XYP1 was dissolved in DMEM. SFZ was purchased from Guangdong Tai Cheng Pharmaceutical Company, Jiangmen, China.

### 2.4. Identification, DNA Sequencing and Bioinformatic Analysis of a Spider Peptide XYP1

Spiders *L*. *coelestis* were collected in Ji’an, Jiangxi Province, China. A full-length cDNA library was generated in accordance with the protocol previously used by our group [16,17]. Multiple sequence alignment was performed using the ClustalX2 program to analyze the amino acid sequence identity of peptide XYP1 with known antimicrobial or anti-*T. gondii* spider peptides, such as Lycosin-I and Lycosin-II (from spider *L. singoriensis*), Lycotoxin II (from spider *Lycosa carolinensis*) and Lycocitin-3 (from spider *L. singoriensis*) [14,18,19,20]. We acquired the helix-wheel plot and analyzed its net charge, hydrophobicity, polar residues + GLY and nonpolar residues by a software package provided by the Expert Protein Analysis System (ExPASy) proteomics server as described in a previous study (https://heliquest.ipmc.cnrs.fr/cgi-bin/ComputParams.py, accessed on 20 March 2019) [21]. The secondary structure was determined by circular dichroism (CD) spectroscopy performed on a J-815 Circular Dichroism Spectropolarimeter (Jasco, Tokyo, Japan). Peptide XYP1 at 100 μmol L^−1^ were analyzed in phosphate-buffered saline (PBS, pH 7.4; HyClone Co., Logan, UT, USA), 50% trifluoroethanol (TFE; Shanghai Macklin Biochemical Co., Shanghai, China), or 100 mmol L^−1^ sodium dodecyl sulfate (SDS; Shanghai Macklin Biochemical Co., Shanghai, China) [22,23]. The spectra were obtained from three separate recordings.

### 2.5. Tachyzoite Mortality

To test the effect of XYP1 on the mortality of *T. gondii* tachyzoites, RH-GFP of tachyzoites (5 × 10^6^) were treated with XYP1 (40, 20, 10, 5, 2.5 μΜ) or DMEM (control group), respectively, for 2 h at room temperature, and then centrifuged at 3000 rpm for 8 min to remove the supernatant. Tachyzoites were then resuspended with PBS and examined by using a Fluorescence Microscope (BZ-9000, Keyence, Tokyo, Japan). Fluorescence intensity represented the viability of parasites: the higher the fluorescence intensity, the lower the tachyzoite mortality and the better the parasitic activity, and vice versa.

### 2.6. Survival Assay

To evaluate the effects of XYP1 on acute *T. gondii* infection in vivo, we used a BALB/c murine model and infected the mice with the RH 2F strain of *T. gondii*. Mice were divided into 3 groups (8 mice/group) and injected intraperitoneally with 10^3^ tachyzoites per mouse. XYP1 and SFZ were dissolved in PBS, respectively. After 4 h of infection, mice were treated for 7 consecutive days with daily intraperitoneal injection of 200 μL PBS, or XYP1 at 4 mg/kg or SFZ at 100 mg/kg (once a day). Mice treated with 200 μL PBS was used as the vehicle. Mice were monitored as described previously [24] and the death dates were recorded.

### 2.7. Invasion Assay

The invasion rate of *T. gondii* was assessed as previously reported [25]. Tachyzoites were incubated with XYP1 (20, 10, 5, 2.5, 1.25 μΜ), SFZ (400 μΜ; positive control) and DMEM (negative control), respectively, at room temperature for 2 h. HFFs cultured in 24-well plates containing 14 mm coverslips were infected with pre-treated tachyzoites with a multiplicity of infection (MOI) of 5 (tachyzoites/cells = 5:1). HFFs were then washed twice with PBS to remove extracellular parasites after 2 h. Afterwards, they were fixed with methanol for 5 min, and then stained with Giemsa (Sigma Chemical Co., Saint Louis, MO, USA) for 20 min. The coverslips were observed under a light microscope (Motic China Group Co., Ltd., Xiamen, China). The invasion rate = the number of tachyzoites in 200 cells/200. Three independent experiments were performed.

### 2.8. Intracellular Proliferation Assay

HFFs (1 × 10^6^) were infected with *T. gondii* tachyzoites with a MOI of 5 for 2 h. Afterwards, monolayers were washed twice with PBS to remove extracellular parasites and treated with XYP1 (20, 10, 5, 2.5, 1.25 μΜ), or SFZ (400 μΜ; positive control), or DMEM (negative control) for 24 h and 48 h, respectively. HFFs were washed twice with PBS, fixed with methanol for 5 min, and then stained by Giemsa for 20 min. These coverslips were observed under the light microscope. Data shown were representative of three independent experiments.

### 2.9. Electron Microscopy Analysis

To further explore the anti-*Toxoplasma* mechanism of XYP1, we observed the ultrastructure of *T. gondii* tachyzoites treated with XYP1 by the scanning electron microscopy (SEM) and transmission electron microscopy (TEM) according to the protocol previously reported [26]. Samples in SEM were observed using a Hitachi S-3400N scanning electron microscope, and in TEM were analyzed using a Tecnai G2 Spirit TWIN transmission electron microscope.

### 2.10. RNA Extraction and RNA-Seq Analysis

Total RNA was extracted from each sample using the mirVana miRNA Isolation Kit (Ambion, New Haven, CT, USA) according to the manufacturer’s protocol. The integrity of all RNA samples was examined using the Agilent 2100 Bioanalyzer (Agilent Technologies, Santa Clara, CA, USA). The samples with RNA Integrity Number (RIN) ≥ 7 were subjected to the subsequent analysis. The libraries were constructed using TruSeq Stranded mRNA LT Sample Prep Kit (Illumina, San Diego, CA, USA), which were then sequenced on the Illumina sequencing platform (HiSeq^TM^ 2500 or Illumina HiSeq X Ten) and 125 bp/150 bp paired-end reads were generated.

Raw data (raw reads) were processed using Trimmomatic [27]. The reads containing poly-N and the low-quality reads were removed to obtain the clean reads, which were mapped to reference genome using hisat2 [28]. StringTie was used to reconstruct the transcripts guided by the genomic annotation information [29]. Novel transcripts were identified using Cufflinks. The coding ability of new transcripts was predicted using Coding Potential Calculator. The high-quality clean reads were then mapped to the reference genomes of *T. gondii* (ftp://ftp.ncbi.nlm.nih.gov/genomes/all/GCA/000/256/705/GCA_000256705.2_TGCAST_v2/GCA_000256705.2_TGCAST_v2_genomic.fna.gz, accessed on 28 February 2020). Fragments per kilobase of exon per million mapped fragments (FPKM) [30] and read counts value of each transcript were calculated using bowtie2 [31] and eXpress [32]. FPKM value of each gene was calculated using cufflinks [33], and the read counts of each gene were obtained by htseq-count [34]. DESeq was used to identify the differentially expressed genes (DEGs) and calculate the *p*-value and fold change. Gene expression with log2 fold change ≥ 0.58 or ≤ −0.58, and adjusted *p*-value < 0.05 was considered as differentially expressed. Kyoto Encyclopedia of Genes and Genomes (KEGG) [35] and Gene Ontology (GO, http://geneontology.org/, accessed on 28 February 2020) were used for pathway annotation and gene enrichment analyses, respectively. The RNA-seq, reads alignment and DEG identification were conducted by OE biotech Co., Ltd. (Shanghai, China).

### 2.11. Quantitative Real-Time PCR

QRT-PCR was used to verify the RNA-seq results. Three *T. gondii* DEGs were randomly selected for qRT-PCR verification and β-actin was used as the reference gene. The RNA samples were reverse-transcribed to single strand cDNA using the PrimeScript^TM^ RT reagent Kit (TaKaRa, Dalian, China). All qRT-PCR reactions were performed on the BIO-CFX96 system (Bio-Rad, Hercules, CA, USA) using SYBR Green GoTaq^®^ qPCR Master Mix (Promega, Beijing, China). The primers used for qRT-PCR are listed in Table S1. The qRT-PCR cycling conditions included 95 °C for 2 min followed by 40 cycles of 95 °C for 10 s, 58 °C for 15 s, 72 °C for 40 s, and the temperatures of the melting curve analysis ranged from 72 to 95 °C. The 2^−ΔΔCq^ method was used to calculate the relative expression of each gene.

### 2.12. Statistical Analysis

The SPSS PASW Statistics version 18.0 (SPSS, Armonk, New York, NY, USA) and GraphPad Prism version 5.0 (GraphPad Software, San Diego, CA, USA) were used to analyze data and construct graphs, respectively.

## 3. Results

### 3.1. Identification and Structural Characteristics of XYP1

**Figure 1:**
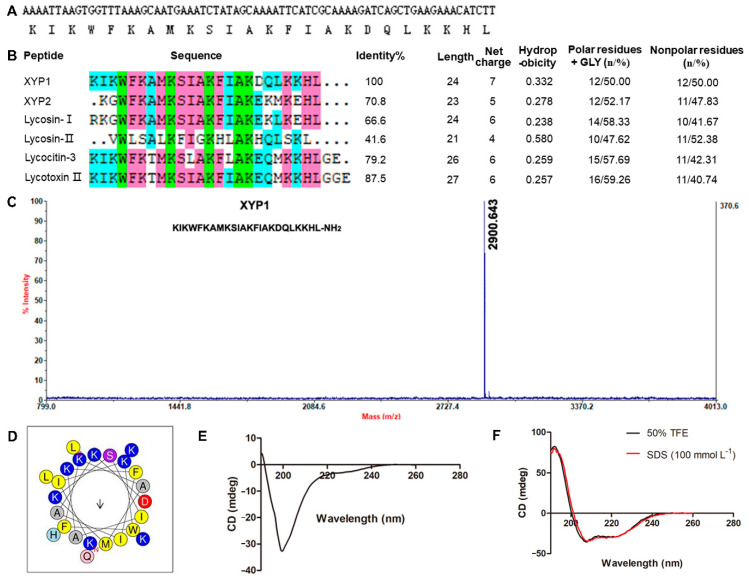
Structure characteristics of XYP1. (**A**) The cDNA sequence and amino acid sequence of XYP1. (**B**) Sequence alignment and physicochemical properties of XYP1 and its analogs. (**C**) Mass spectra of XYP1. The calculated molecular weight of XYP1 was 2901.64 Da and the measured molecular weight was 2900.643 Da. (**D**) Helical wheel projection diagrams of XYP1. The hydrophobic residues are presented in yellow color, positively charged hydrophilic residues blue, the noncharged polar residue purple, and negatively charged hydrophilic residue red. The circular dichroism spectra of XYP1: in PBS (pH 7.4) (**E**) and in the presence of 50% TFE or SDS (100 mmol L^−^^1^) (**F**) at 25 °C.

By establishing the cDNA library of venom gland of spider *L. coelestis*, we cloned a new gene, and its encoded peptide was named XYP1. It composes of 24 amino acids and its amino acid sequence is KIKWFKAMKSIAKFIAKDQLKKHL (Figure 1A). Bioinformatics analysis demonstrated that XYP1 has 7 net charges with theoretical isoelectric point of 10.48, hydrophobicity of 0.332, 50% non-polar amino acid residues, and its sequence identity with peptide XYP2 (from spider *Lycosa sinensis*), Lycosin-I (from spider *L. singoriensis*), Lycotoxin II (from spider *Lycosa carolinensis*) and Lycocitin-3 (from spider *L. singoriensis*) are 70.8%, 66.6%, 87.5% and 79.2%, respectively (Figure 1B). The measured monoisotopic molecular weight of XYP1 (2900.643 Da) is slightly lower than the calculated mass (2901.64 Da) (Figure 1C), indicating that it bears an amidated carboxyl terminus. The property of α-helix in the protein or peptide can be observed in the wheel projection [36]. Hydrophobic amino acids are concentrated on one side of the helix, and polar or hydrophilic amino acids are located on the other side [37]. According to the wheel projection, XYP1 possessed hydrophobic phenylalanine (Phe, F), isoleucine (Ile, I), alanine (Ala, A), methionine (Met, M) and tryptophan (Trp, W) residues on the hydrophobic side, while the hydrophilic lysine (Lys, K) and aspartate (Asp, D) residue and the polar serine (Ser, S) residue were observed on the opposite side (Figure 1D). Helical wheel projection diagrams of XYP1 draw the amino acid sequence constituting the helical region of the peptide secondary structure in a rotating manner (Figure 1D). To further verify that XYP1 adopted an α-helix structure, circular dichroism (CD) spectroscopy was performed and the results revealed that XYP1 adopted a random coil structure in PBS (Figure 1E), but an α-helix structure in 50% TFE and SDS (100 mmol L^−1^) (Figure 1F). These results suggested that XYP1 was a dipolar molecule that could form an amphipathic helical structure in an appropriate environment. According to the previous study, the amphiphilic α-helical structure could help peptides to be inserted into the cell membrane, thereby destroying the integrity of the cell membrane and resulting in leakage of the cell contents and the imbalance of homeostasis [38], which reminds us that XYP1 may also have this function.

### 3.2. XYP1 Exhibits Potent Anti-Toxoplasma Activity In Vitro and In Vivo

To verify the anti-*T*. *gondii* potential of XYP1, we evaluated the efficacy of XYP1 against *T. gondii* tachyzoites by a fluorescence microscope and the survival assay.

**Figure 2:**
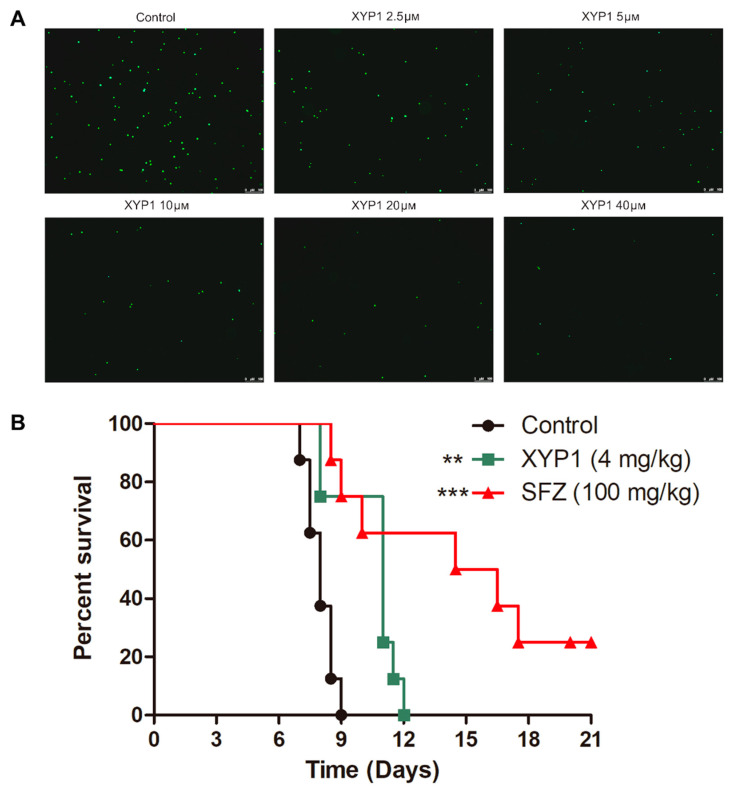
Anti-*T*. *gondii* effects of XYP1 in vitro and in vivo. (**A**) Viability assessment of *T. gondii* tachyzoites evaluated by fluorescence microscope. RH-GFP of tachyzoites were treated with different concentrations of XYP1 or DMEM alone (control) for 2 h. The fluorescence intensity was observed using a fluorescence microscope. *Scale bars* = 100 μm. (**B**) Effect of XYP1 and SFZ on the survival rate of infected mice. RH 2F strain tachyzoites. Beginning on the day of infection, XYP1 at 4 mg/kg, vehicle (PBS) or positive drug (SFZ) at 100 mg/kg was intraperitoneally administered for 7 days. These mice were observed for an additional 14 days, and the survival times of the infected mice were recorded for 21 days (*n* = 8 for each group). A log-rank test demonstrated that SFZ and XYP1 statistically prolonged the survival time of infected mice compared with the vehicle group (*** *p* < 0.0001, ** *p* < 0.01).

Based on the fluorescence intensity, we observed that XYP1 had significant anti-*T. gondii* activities at the concentration of 2.5 μΜ, and the fluorescence could hardly be seen when the concentration of XYP1 was 20 or 40 μΜ (Figure 2A). The results showed that XYP1 treatment significantly reduced extracellular *T. gondii* tachyzoites viability and enhanced their mortality.

Due to the good anti-*Toxoplasma* activity of the peptide XYP1 in vitro, we next determined the anti-*Toxoplasma* effects of XYP1 in mice acutely infected by the RH strain of *T. gondii*. A log-rank test demonstrated a statistically significant difference in survival rates between mice treated with XYP1 and the vehicle (*p* < 0.01), as well as mice treated with SFZ and the vehicle (*p* < 0.0001) (Figure 2B). In comparison to the median survival of the vehicle (8 days), mice treated with XYP1 (11.75 days) had a significantly longer survival time.

### 3.3. XYP1 Suppresses Invasion of T. gondii Tachyzoites into Host Cells

**Figure 3:**
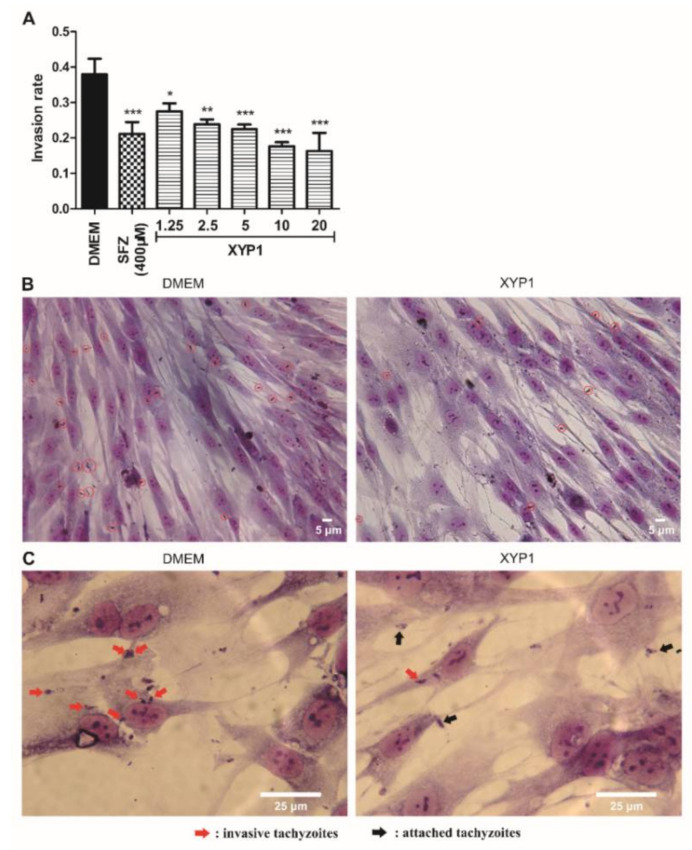
Effects of XYP1 on the invasion of *T. gondii* tachyzoites. Tachyzoites were pre-treated with DMEM (the negative control), SFZ (the positive control), and two-fold serial dilutions of XYP1 before exposure to host cells, respectively. Statistical results were expressed as the invasion rate (**A**). HFFs in DMEM group and XYP1 group (10 μΜ) were observed by a light microscope (40×) (**B**). HFFs in DMEM group and XYP1 group (10 μΜ) were also observed by a light microscope (100×) (**C**). The means were determined by values obtained from three independent experiments (χ^2^-tests). * *p* < 0.05, ** *p* < 0.01 and *** *p* < 0.001 in comparison with control. *Scale bars* = 5 μm (**B**); 25 μm (**C**).

*T. gondii* tachyzoites pre-treated with DMEM or SFZ showed an invasion rate of 38% and 21.17%, respectively, while pre-treatment with XYP1 decreased its invasion rate to 27.5, 23.83, 22.5, 17.67 and 16.33% at the concentration of 1.25, 2.5, 5, 10 and 20 μΜ, respectively (Figure 3). The inhibition effect of XYP1 on tachyzoite invasion rate was found to be concentration-dependent, and the invasion rate in XYP1 (20 μΜ) group was decreased by 57.03% ((38% − 16.33%)/38%) compared with that in the control group. Parasites in the PVs could be clearly seen in the control group, whereas parasites treated with XYP1 (10 μΜ) only attached to membranes of the host cells. Additionally, it is clear that there are more invasive tachyzoites in the control group than in the XYP1-treated group (Figure 3B red circles and Figure 3C red arrows). The results indicated that the invasion ability of *T. gondii* tachyzoites treated with different concentrations of XYP1 was significantly reduced compared with that of untreated ones (*p* < 0.05).

### 3.4. XYP1 Inhibits Intracellular Proliferation of T. gondii Tachyzoites

**Figure 4:**
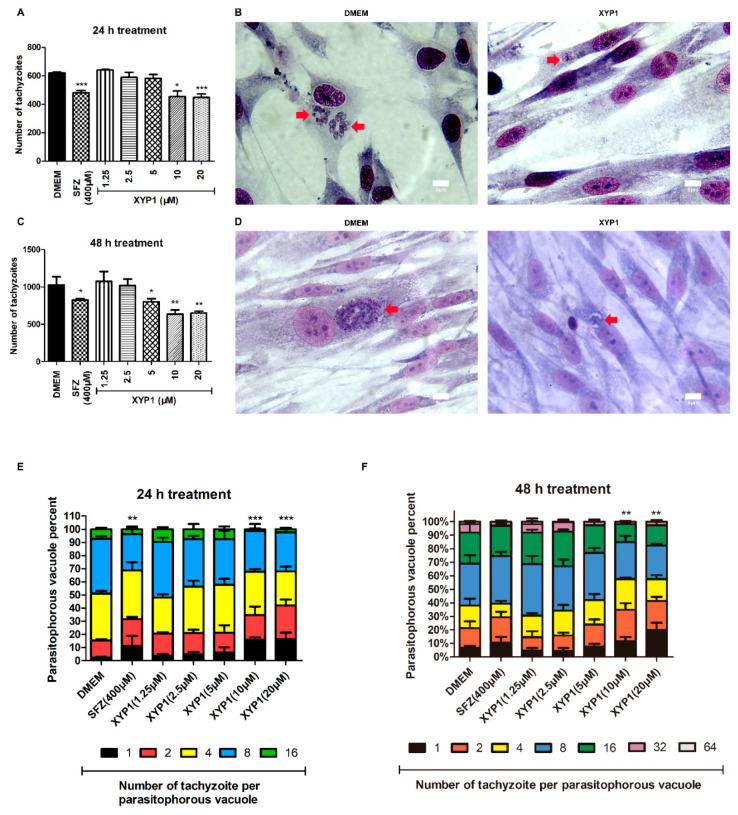
Effects of XYP1 on the proliferation of *T. gondii* tachyzoites. HFFs were infected with tachyzoites and treated with two-fold serial dilutions of XYP1 and SFZ for 24 (**A**) or 48 h (**C**) to evaluate their effects on the tachyzoite proliferation. Infected cells were incubated with DMEM or XYP1 for 24 h (**B**) or 48 h (**D**), and then observed by the light microscope. The number of tachyzoites per PV was also calculated to evaluate their anti-tachyzoite effects (**E**,**F**). The means were determined by values obtained from three independent experiments. * *p* < 0.05, ** *p* < 0.01 and *** *p* < 0.001 in comparison with negative control. *Scale bars* = 5 μm.

In the 24 h-treatment group, compared with tachyzoites in the negative control, SFZ (400 μΜ) and XYP1 (5, 10, or 20 μΜ) decreased the proliferation rate by 22.65, 6.33, 26.78 and 27.91%, respectively (*p* < 0.05, *p* < 0.001) (Figure 4A). As for the 48 h-treatment group, the proliferation rate was decreased by 38.04% and 36.71% when treated with XYP1 (10 or 20 μΜ) compared with the negative control (*p* < 0.01) (Figure 4C).

Most of the PVs in the control group contained over sixteen parasites and formed typical rosettes, while PVs in the XYP1 (10 μΜ) groups contained fewer parasites (Figure 4B,D). After 24 h treatment, the minimum percentage of PVs containing 16 tachyzoites appeared in XYP1 (10 μΜ) group, while the percentage of PVs with 16 tachyzoites in DMEM-treated group was quite high (Figure 4E). After 48 h treatment, the percentage of PVs containing 64 or 32 tachyzoites in SFZ or XYP1 (5, 10, 20 μΜ) groups was significantly reduced compared with that in the control group. Note that there were no PVs containing 64 tachyzoites in SFZ or XYP1 (5, 10, 20 μΜ) groups (Figure 4F). Moreover, the maximum percentage of PVs with only 1 tachyzoite was in the XYP1 (20 μΜ) group both after 24 h and 48 h treatment. Therefore, compared with that in the control group, the percentage of PVs containing more tachyzoites (16, 32 and 64) was significantly decreased by XYP1 (10, 20 μΜ) within 24 h (*p* < 0.001) (Figure 4E). These results indicated that the proliferation of *T. gondii* tachyzoites was significantly inhibited by XYP1, and the inhibition rate was concentration-dependent.

### 3.5. XYP1 Causes Membrane Perforation, Swelling and Disruption of T. gondii Tachyzoites

**Figure 5:**
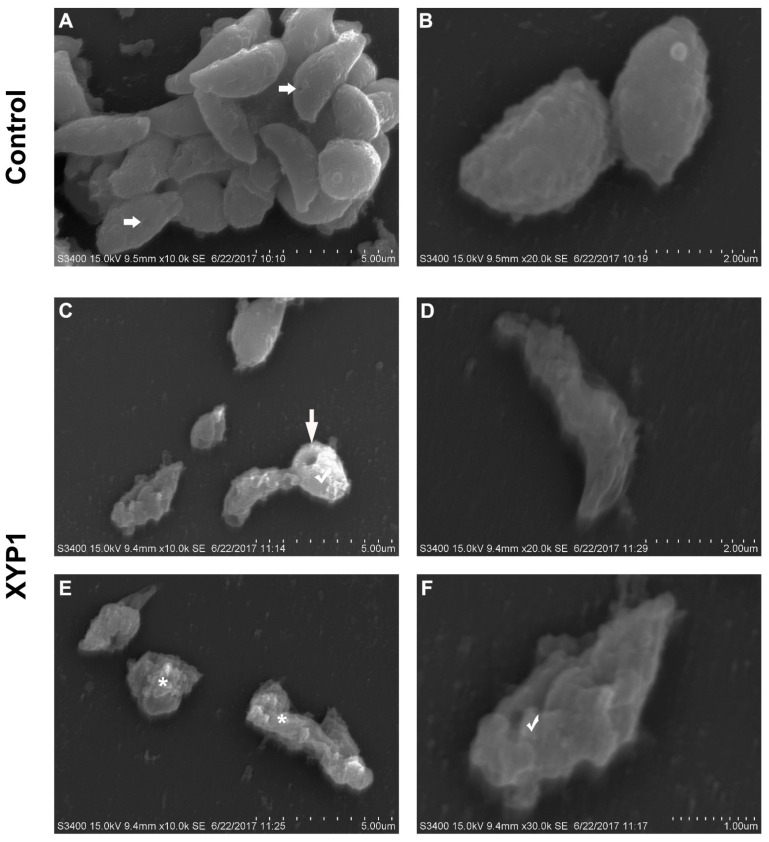
*Morphological* changes of *T. gondii* tachyzoites treated with XYP1 as visualized by SEM. Parasites treated with DMEM were defined as the negative control (**A**,**B**). Parasites were exposed to 10 μM XYP1 for 2 h (**C**–**F**). Markers: horizontal arrows (→) in A: primary portal of tachyzoites; vertical arrow (↓) in C: hole of tachyzoites; asterisk (*) in E: disintegrated tachyzoites; hooks (√) in c and F: swelled tachyzoites. *Scale bars* = 5 μm (**A**,**C**,**E**); 2 μm (**B**,**D**); 1 μm (**F**).

**Figure 6:**
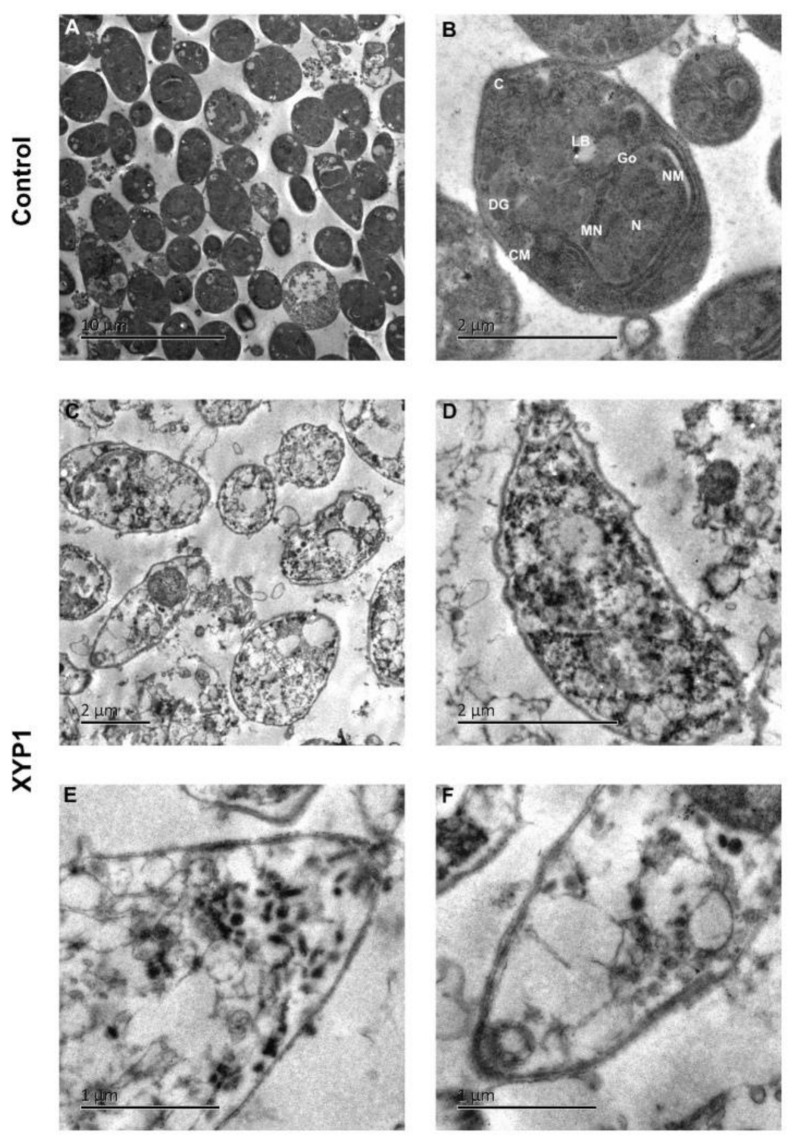
Ultrastructural effects of XYP1 on *T. gondii* tachyzoites as visualized by TEM. Parasites treated with DMEM were defined as the negative control (**A**,**B**). Tachyzoites were exposed to 10 μM XYP1 for 2 h (**C**–**F**). Abbreviations in B: C, conoid; CM, cell membrane; DG, dense granule; Go, golgi complex; LB, lipid body; MN, microneme; N, nucleus; NM, nuclear membrane. *Scale bars* = 10 μm (**A**); 2 μm (**B**–**D**); 1 μm (**E**,**F**).

SEM was employed to assess the morphological changes of tachyzoites caused by XYP1 treatment. In the control group, tachyzoites appeared to typically be crescent shaped with a pointed head (conoid), a blunt tail and a micropore; moreover, their surfaces were smooth, regular and complete. The micropore, located in the middle of the tachyzoite body, is considered as the primary portal where endocytosis takes place (Figure 5A,B; horizontal arrows). Obviously, most of tachyzoites treated with XYP1 (10 μΜ) lost the crescent shape with deep dimples, holes and wrinkles on the surfaces (Figure 5C–F; vertical arrow). Some tachyzoites swelled up (Figure 5C,F; hooks), or even completely collapsed and disintegrated with plasmoptysis (Figure 6E; asterisk).

The longitudinal and transverse sections of tachyzoites were observed by photomicrographs of TEM (Figure 6A). The tachyzoites in the control group maintained their normal biological features with arranged and integrated organelles (Figure 6B). However, the tachyzoites treated with XYP1 showed malformation and their distribution of organelles was altered. The condensed chromatin and cytoplasm vacuolization were observed in XYP1-treated group (Figure 6C). Most of tachyzoites largely lost their biological features with organelles disappeared and nuclear membranes destroyed (Figure 6D–F).

Next, morphological analysis was also conducted using AO/PI dual staining assay and examined under the fluorescence microscope. The results showed the representative image of AO/PI staining of GFP-TgAtg8 strain tachyzoites treated with different concentrations of XYP1 (Figure S3). In the control group, the tachyzoites remained uniformly stained with complete membrane and normal morphological structure, and after 2 h, there were large numbers of viable tachyzoites (green color) and very few apoptotic ones (yellow to orange color). The XYP1-treated group showed a large number of necrotic tachyzoites (bright red color) and few live tachyzoites (Figure S3A). As seen in Figure S3B, there is a strong dose–response relationship in regard to XYP1 treatment, showing a significant increase in the percentage of dead tachyzoites compared to that in the control group (*** *p* < 0.0001).

### 3.6. Transcriptome Profiling Reveals the Underlying Mechanisms of Action of XYP1

**Figure 7:**
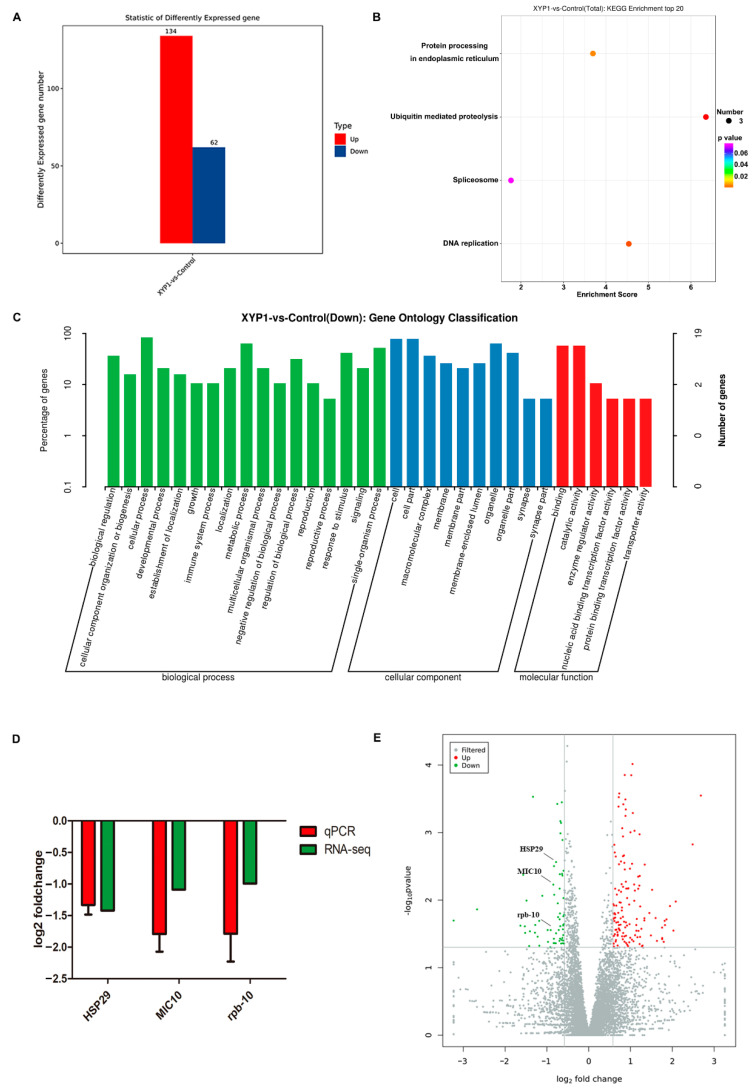
Gene expression profiles after XYP1 treatment and verification of the RNA-seq data by qRT-PCR. (**A**) The numbers of *T. gondii* DEGs after 8 h XYP1 treatment. (**B**) KEGG pathway enrichment. (**C**) GO classification. (**D**) Verification of the RNA-seq data by using qRT-PCR. (**E**) Volcano plot of DEGs. Significantly upregulated and downregulated genes are marked with red and blue spots, respectively.

RNA-seq was performed to characterize the expression patterns of genes in *T. gondii* tachyzoites after XYP1 treatment, and the results showed that a series of genes were differentially expressed in the 8 h XYP1-treatment tachyzoites (Figure S5), in which there were 134 upregulated and 62 downregulated genes (Figure 7A). According to the KEGG enrichment, XYP1 mainly regulated four pathways, including protein processing in endoplasmic reticulum, ubiquitin meditated proteolysis, spliceosome and DNA replication. Micronemes can secrete microneme proteins, which are synthesized in the endoplasmic reticulum. MIC10, related to the growth and proliferation of *T*. *gondii* tachyzoites [39], was downregulated by XYP1 according to RNA-seq. Previous assays (SEM and AO/PI staining) suggested that the membrane of *T*. *gondii* tachyzoites was destroyed by XYP1, and according to the GO enrichment, XYP1 downregulated genes related to the membrane and membrane part. HSP29 is an important component of the membrane of *T*. *gondii* and may be a potential anti-*Toxoplasma* drug target [40]. The coordinated control of gene expression is undertaken at the level of transcription of specific mRNAs by RNA polymerase II (RNAPII), in a multistep, sequential pattern with distinct phases: preinitiation, initiation, promoter clearance, elongation, RNA processing, and termination [41]. Rpb-10, belonging to RNAPII, was downregulated by XYP1, which may account for the downregulated transcription level of *T. gondii* tachyzoites. The expression of three *T. gondii* genes (MIC10, HSP29 and rpb-10) obtained through RNA-seq were confirmed by qRT-PCR and the validation results are shown in Figure 7D. Therefore, XYP1 may target some key targets, inhibiting the viability, growth, invasion, and proliferation of *T. gondii* tachyzoites.

## 4. Discussion

*T. gondii*, an opportunistic parasite, affects approximately 30% of the world’s population and can be fatal in immunologically compromised individuals. Conventional drugs with side effects necessitate the development of new drugs that can target *T. gondii* more specifically and efficiently [5,7].

In this study, 5 μM XYP1 achieved remarkable efficacy against RH-GFP *T. gondii* tachyzoites (Figure 2A) with low cytotoxic effects on HFFs under 20 μΜ (IC_50_ = 38.79 μΜ) (Figure S1A), which meant that the concentration we chose (10 μΜ) had nearly no cytotoxicity on HFFs but might be effective enough to kill *T. gondii* tachyzoites. Hemolysis is a common side effect of peptides against mammalian cells. Lycosin-I and Lycosin-II, two linear cationic α-helical peptides derived from the venom of the spider *L. singoriensis*, led to approximately 10% and 20% hemolysis, respectively, at the concentration of 50 μM [18,42], while XYP1 showed nearly no hemolysis at the range of 1.25 to 80 μΜ and induced only 6.15% of hemolysis even at the highest concentration (160 μM) (Figure S1B). Compared with both Lycosin-I and Lycosin-II, XYP1 was much safer and could possibly be a reliable anti-*T. gondii* candidate drug. XYP1 could not only effectively inhibit the invasion and proliferation of *T. gondii* in a dose-dependent manner with low cytotoxicity against HFFs, but also significantly prolong the survival time of mice acutely infected with *T. gondii*, indicating that XYP1 has potent anti-*Toxoplasma* activity in vitro and in vivo. However, the average survival time of XYP1 (4 mg/kg)-treated mice was shorter than that of SFZ (100 mg/kg)-treated ones. Therefore, the treatment conditions of XYP1, such as the dose, time and route of administration, need to be further optimized. 

Phospholipids on cell membranes are important targets of active peptides with α-helical structure [43]. The side chains of positively charged amino acids interact with the negatively charged phospholipids on the cell membrane through electrostatic interaction to form an amphiphilic α-helical structure on the cell membrane surface, which helps peptides to be inserted into the cell membrane, thereby destroying the integrity of the cell membrane and resulting in leakage of the cell contents and the imbalance of homeostasis [38]. We confirmed that XYP1 can form an α-helical structure through CD spectroscopy (Figure 1E,F). SEM and TEM analysis showed that XYP1 significantly destructed the tachyzoite structure, resulting in membrane perforation, swelled appearance, plasmoptysis, and alterations in the organelle distribution (Figure 5 and Figure 6). It was also shown that XYP1 disrupted the permeability and integrity of the tachyzoite membrane (Figure S3). Therefore, we speculated that XYP1 could form pores by targeting the phospholipids of the membrane of *T*. *gondii*, increasing the permeability of its membrane and destroying its integrity, which in turn kills *T*. *gondii*. 

**Figure 8:**
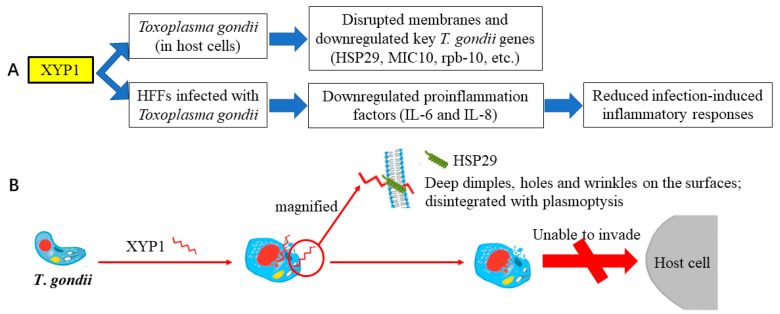
Proposed mechanism of XYP1. (**A**) Proposed mechanism of the anti-*T*. *gondii* action and protective effect on HFFs of XYP1. (**B**) Proposed mechanism of the anti-*T*. *gondii* action. XYP1 may downregulate the expression of HSP29, and consequently disrupt the tachyzoite membrane and inhibit its invasion and proliferation.

RNA-seq and qRT-PCR verification showed that XYP1 significantly downregulated the mRNA expression levels of HSP29, MIC10 and rpb-10 of *T*. *gondii* (Figure 7). HSP29 is an important component of the membrane of *T*. *gondii* and may be a potential anti-*Toxoplasma* drug target [40]. The primary structure analysis of HSP29 showed that there is an abnormal segment rich in threonine residues in the N-terminal region, and there is a putative transmembrane domain between amino acids 125 and 146 [40]. Therefore, we proposed an underlying mechanism by which XYP1 could disrupt the tachyzoite membrane and inhibit its invasion and proliferation through downregulating the expression of HSP29 (Figure 8B). However, the hypothesis needs to be further investigated and proved.

Previous studies have identified the critical role of IL-6 in inhibiting tachyzoite proliferation and toxoplasmic encephalitis. Additionally, the release of IL-8 by *T. gondii*-infected cells could recruit effector cells against parasites [44]. IL-6 and IL-8, two important inflammatory factors for hosts to defend against *T. gondii*, were significantly increased in the host cells following challenge with *T. gondii* compared with the control group (Figure S4). In addition, high expression of inflammatory factors may mediate the host pathology [45]. Additionally, there was a significant reduction of the two inflammatory factors in the XYP1-treated group compared with those of the control group (Figure S4). Taken together, XYP1 may relieve the host pathology caused by *T. gondii* and moderately regulate the expression of the two inflammatory factors to resist *T. gondii* (Figure 8A).

Although we provided evidence that the peptide XYP1 could act as a promising anti-parasitic drug, there are also some limitations. In the plaque assay, no plaque could be seen by the naked eye in SFZ-treated group (Figure S2). We guessed SFZ might be more stable or have a longer half-life than XYP1, while according to the invasion and proliferation assays, XYP1 was more effective than SFZ within 48 h. Hence, if we could stabilize XYP1 or prolong its half-life, it would be a potential therapeutic candidate in the future. Therefore, we will try to figure out the anti-*T. gondii* mechanism of XYP1 and modify its structure to improve its anti-*T. gondii* activity and stability in hosts in upcoming studies. We will employ several approaches to address the limitations of XYP1. In a previous study, we studied the anti-*T. gondii* effects of Lycosin-I [14], and similarly, in the present study, we found the amino acid sequence of XYP1 shares a 66.6% identity with that of Lycosin-I, most of which are hydrophobic amino acids (leucine, isoleucine, alanine, methionine, etc.) and positively charged hydrophilic residues (lysine). These suggested that the anti-*T. gondii* effects of XYP1 may depend on some basic amino acid residues. Therefore, we will determine the functional amino acid residues of XYP1 and modify those non-functional ones to improve its anti-*T. gondii* activities and decrease its cytotoxic and hemolytic activities. We may also apply fatty acid modification to improve its stability, hoping that we can develop a modified peptide that can be expected to serve as a promising anti-*T. gondii* drug based on XYP1.

## 5. Conclusions

Considering the side effects and drug resistance of previous anti-*T. gondii* drugs or compounds, new therapeutic strategies for toxoplasmosis are urgently needed. Peptide XYP1 derived from the spider venom showed anti-*T. gondii* activity, protective immunity, low cytotoxicity and hemolysis to the host, making it a promising anti-parasitic compound.

## Data Availability

All data generated or analyzed during this study are included in this manuscript and its supplementary information file.

## Supplementary Materials

The following are available online at https://www.mdpi.com/2227-9059/9/8/934/s1.

## Abbreviations

AO/PI	acridine orange/propidium iodide
CD	circular dichroism
cDNA	complementary DNA
DEGs	differentially expressed genes
DMEM	Dulbecco’s modified Eagle’s medium
DMSO	dimethyl sulfoxide
FBS	fetal bovine serum
FPKM	fragments per kilobase of exon per million mapped fragments
HFFs	human foreskin fibroblasts
IL-6	interleukin-6
IL-8	interleukin-8
*L. coelestis*	*Lycosa coelestis*
*L. singoriensis*	*Lycosa singoriensis*
MTT	methyl thiazolyl tetrazolium
PBS	phosphate-buffered saline
qRT-PCR	quantitative real-time PCR
RBCs	red blood cells
RNA-seq	RNA sequencing
SDS	sodium dodecyl sulfate
SEM	scanning electron microscopy
SFZ	sulfadiazine
SPF	specific-pathogen-free
TEM	transmission electron microscopy
TFE	trifluoroethanol
*T. gondii*	*Toxoplasma gondii*
