# Correction: Liu et al. Anti-*Toxoplasma gondii* Effects of a Novel Spider Peptide XYP1 In Vitro and In Vivo. *Biomedicines* 2021, *9*, 934

**DOI:** 10.3390/biomedicines10051176

**Published:** 2022-05-19

**Authors:** Yuan Liu, Yaqin Tang, Xing Tang, Mengqi Wu, Shengjie Hou, Xiaohua Liu, Jing Li, Meichun Deng, Shuaiqin Huang, Liping Jiang

**Affiliations:** 1 Department of Parasitology, Xiangya School of Medicine, Central South University, Changsha 410013, China; 186511077@csu.edu.cn (Y.L.); hanyin998@csu.edu.cn (Y.T.); wumengqi@csu.edu.cn (M.W.); hsj1199@csu.edu.cn (S.H.); liuxiaohua0306@csu.edu.cn (X.L.); lijing0807@csu.edu.cn (J.L.); huangshuaiqin@xmu.edu.cn (S.H.); 2 Hunan Key Laboratory for Conservation and Utilization of Biological Resources in the Nanyue Mountainous Region, Hengyang Normal University, Hengyang 421008, China; xtang2011@sina.com; 3 Department of Biochemistry and Molecular Biology, School of Life Sciences, Central South University, Changsha 410013, China; dengmch@csu.edu.cn; 4 China-Africa Research Center of Infectious Diseases, Xiangya School of Medicine, Central South University, Changsha 410013, China

In the original publication, there were mistakes in Figure 3C, Figure 6B and Figure S2A,B as published [1]. Due to the careless combination of images, incorrect images were inadvertently inserted in Figure 3C (the magnified image of XYP1 group), Figure 6B (the magnified image of the control group; tachyzoite with the blunt head and tail) and the SFZ group and the Cell group in Figure S2A,B. The corrected Figure 3, Figure 6 and Figure S2 appears below. The authors apologize for any inconvenience caused and state that the scientific conclusions are unaffected. The original publication has also been updated.

There was an error in the original publication. According to the correction to Figure 3C, a correction has been made to the second last sentence of the paragraph in Section 3.3. XYP1 Suppresses Invasion of *T. gondii* Tachyzoites into the Host Cells: “Additionally, it is clear that there are more invasive tachyzoites in the control group than in the XYP1-treated group (Figure 3B red circles and Figure 3C red arrows)”.

The authors apologize for any inconvenience caused and state that the scientific conclusions are unaffected. The original publication has also been updated.

## Figures and Tables

**Figure 3:**
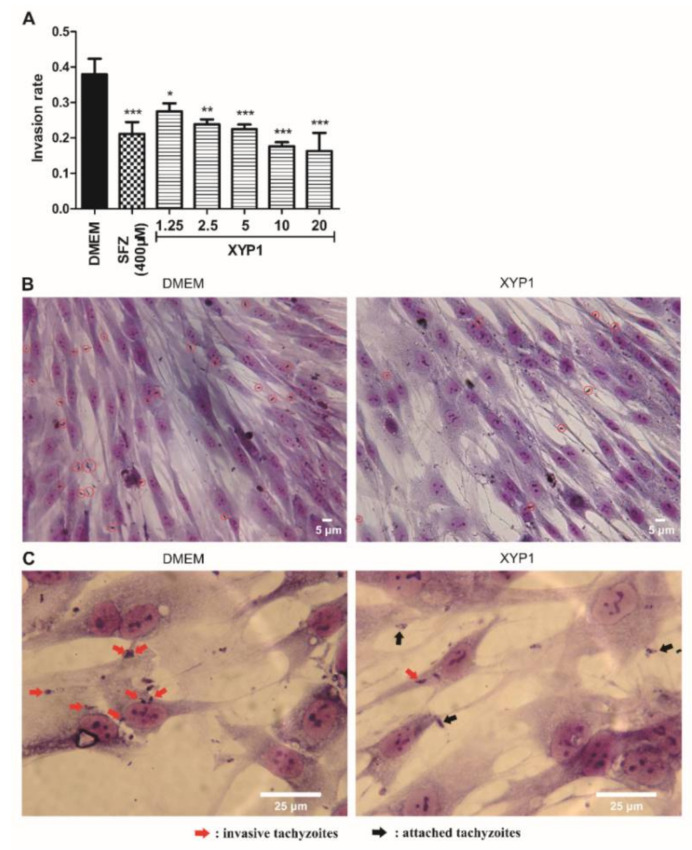
Effects of XYP1 on the invasion of *T. gondii* tachyzoites. Tachyzoites were pre-treated with DMEM (the negative control), SFZ (the positive control), and two-fold serial dilutions of XYP1 before exposure to host cells, respectively. Statistical results were expressed as the invasion rate (**A**). HFFs in DMEM group and XYP1 group (10 μΜ) were observed by a light microscope (40×) (**B**). HFFs in DMEM group and XYP1 group (10 μΜ) were also observed by a light microscope (100×) (**C**). The means were determined by values obtained from three independent experiments (χ^2^-tests). * *p* < 0.05, ** *p* < 0.01 and *** *p* < 0.001 in comparison with control. Scale bars = 5 μm (**B**); 25 μm (**C**).

**Figure 6:**
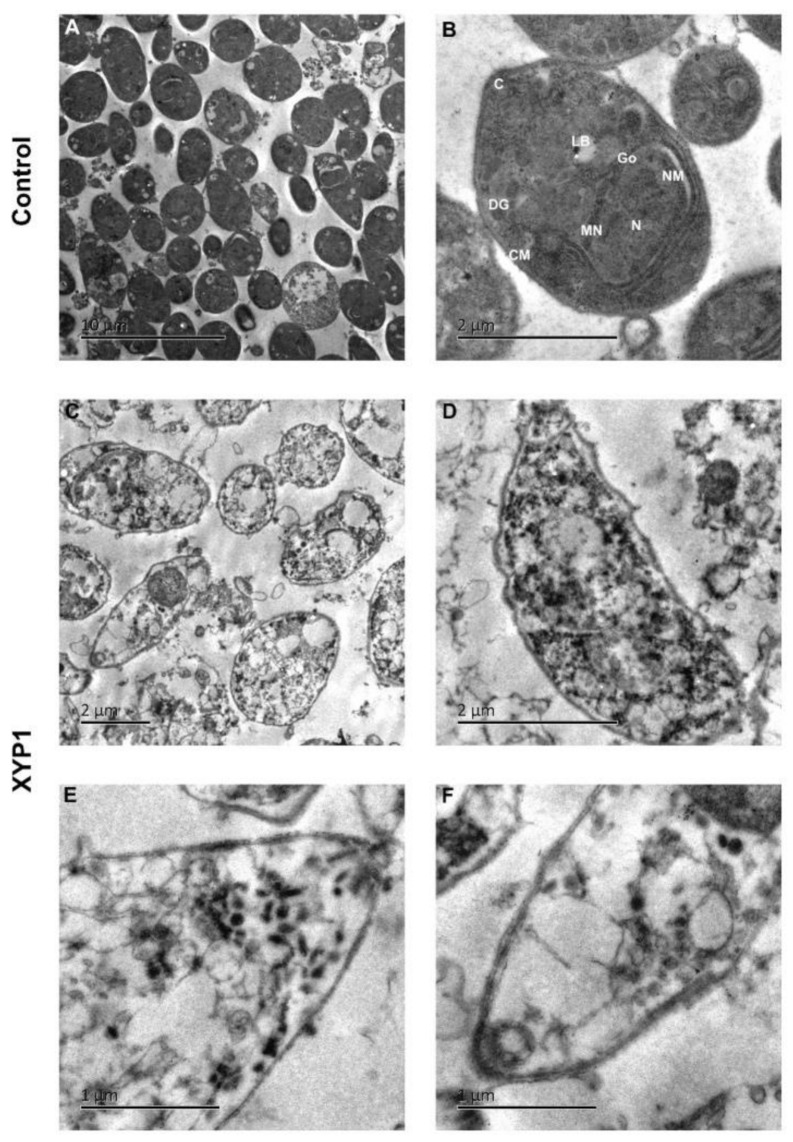
Ultrastructural effects of XYP1 on *T. gondii* tachyzoites as visualized by TEM. Parasites treated with DMEM were defined as the negative control (**A**,**B**). Tachyzoites were exposed to 10 μM XYP1 for 2 h (**C**–**F**). Abbreviations in B: C, conoid; CM, cell membrane; DG, dense granule; Go, golgi complex; LB, lipid body; MN, microneme; N, nucleus; NM, nuclear membrane. Scale bars = 10 μm (**A**); 2 μm (**B**–**D**); 1 μm (**E**,**F**).

**Figure S2:**
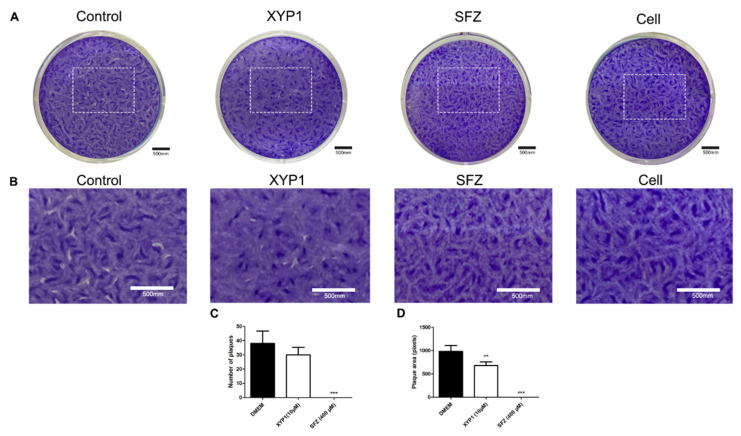
Plaque assay. HFFs were infected with tachyzoites and treated with DMEM (negative control), XYP1 (10 μΜ) and SFZ (positive control, 400 μΜ), respectively, for 7 days. (**A**) Photos of representative wells. (**B**) Magnified zones of plaques graphs. (**C**) The number of plaques determined manually. (**D**) Plaque area determined by Photoshop CS7. The means were determined by values obtained from three independent experiments (Student’s two-tailed *t* test). ** *p* < 0.01 and *** *p* < 0.001 compared with the negative control. Scale bars = 500 mm.
