# Supplementary material for: Anti-Toxoplasma gondii Effects of a Novel Spider Peptide XYP1 In Vitro and In Vivo
Source: Biomedicines. 2021 Aug 1;9(8):934. doi: 10.3390/biomedicines9080934 (PMC8392294; doi:10.3390/biomedicines9080934)
Supplement: Supplementary file 1 [file biomedicines-09-00934-s001.zip › biomedicines-1299859-supplementary materials.pdf]

Supplementary Material

Anti-*Toxoplasma gondii* Effects of a Novel Spider Peptide XYP1 In Vitro and In Vivo

Hemolysis and cytotoxicity assays

To evaluate possible side effects of XYP1, human red blood hemolysis and cytotoxicity assays were performed as described in previous reports with minimum modifications [15,17]. For hemolysis assays, normal human red blood cells (RBCs) were centrifuged, washed three times with PBS until there was no evident red in the supernatant, and then resuspended in PBS. Next, 100 μL of XYP1 solution (160, 80, 40, 20, 10, 5, 2.5, 1.25 μM) was added to 100 μL RBCs in sterile 96-well plates, which were then incubated for 30 min at 37 °C and centrifuged at 1000 rpm for 5 min. Aliquots (100 μL) of supernatant were transferred to sterile 96-well plates, and the hemoglobin release was measured using a Microplate Reader by absorbance at 540 nm (Abs 540 nm). The value for zero and 100% hemolysis was determined using sterile PBS and 1% Triton X-100, respectively. The percentage of hemolysis (%) was calculated as [(Abs 540 nm in the XYP1 solution–Abs 540 nm in PBS) / (Abs 540 nm in 1% Triton X-100–Abs 540 nm in PBS)] × 100%.

In addition, the methyl thiazolyl tetrazolium (MTT; Sigma Chemical Co., Saint Louis, MO, USA) assay was performed to evaluate the cytotoxicity of XYP1 to HFFs as described in previous researches [37]. Briefly, HFFs were cultured in 96-well plates (1× 10^4 cells/well) with 100 μL complete DMEM medium each well for 24 h. HFFs were then treated with two-fold serial dilutions of XYP1 (160, 80, 40, 20, 10, 5, 2.5, 1.25 μM) for 24 h. HFFs incubated with only 100 μL complete DMEM medium each well were regarded as the control. Afterwards, HFFs in 96-well plates were washed with PBS, and 10% MTT dissolved in 100 μL complete DMEM medium were added to each well for 4 h at 37 °C. Finally, the supernatant was removed, and formazan crystals were solubilized in DMSO (100 μL) for 10 min. The optical density was read in a plate reader at 570 nm. The results represented the inhibition viability of XYP1 to HFFs. This assay was performed three times in triplicate. The median toxic concentration (TC_{50}) of XYP1 to HFFs was determined using nonlinear regression analysis by GraphPad Prism 5 (GraphPad Software Inc., San Diego, CA, USA). The inhibition rate of HFFs (%) was calculated as 1 − [(the absorbance of cells treated with the extracts / (the absorbance of cells cultured with medium alone) × 100%].

Plaque assay

HFFs in 6-well plates were infected with fresh tachyzoites (500 tachyzoites/well). After 2 h, HFFs were treated with XYP1 (10 μM), SFZ (400 μM; positive control), and DMEM (negative control), respectively. After 7 days, HFFs were washed three times with PBS, fixed with methanol for 5 min and dyed with crystal violet (0.1%, Sigma Chemical Co., Saint Louis, Missouri, USA) for 20 min. Plates were emptied, washed, air-dried, and photographed using a digital camera (Nikon Corp, Tokyo, Japan). The number of plaques was counted manually and the plaque area was quantified by using Adobe Photoshop CS6 (64 Bit) (Adobe Systems Inc., San Jose, CA, USA).

Acridine Orange and Propidium Iodide (AO/PI) double staining

Acridine orange/propidium iodide (AO/PI) is a rapid, sensitive, and effective method to examine cellular morphology, live and dead cells, apoptosis and/or necrosis. In the present study, AO/PI was used to study the mode of cell death and the effects of XYP1 on the membrane of *T. gondii* tachyzoites by following the method of previous studies with slight modifications. *T. gondii* tachyzoites were treated with different concentrations of XYP1 for 2 h. Tachyzoites without XYP1 treatment were used as a control. After incubation, tachyzoites were harvested and washed with PBS twice. Then, pellets were suspended in 50 μL of acridine orange solution (10 μg/mL) and 50 μL of propidium iodide solution (10 μg/ mL) for 10 min. A volume of 10 μL of stained tachyzoites was dropped to glass slide and covered with a cover slip. Viable and necrotic cells were observed using the fluorescence microscope. This experiment was done in triplicate.

Measurement of cytokine production

To determine the effect of XYP1 regarding immunomodulatory action and evaluate the immune response of host cells after *T. gondii* infection and XYP1 treatment, the expression of two cytokines was measured by qRT-PCR assay. To

prepare the RNA, HFFs were cultured with complete DMEM in 6-well plates and infected with *T. gondii* tachyzoites (MOI = 5) for 2 h when HFFs became 80% confluent. Next, they were treated with XYP1 (10 μM) or DMEM alone (control) and incubated at 37 °C and 5% CO_2 for 48 h. Total RNA was extracted using the Trizol (Invitrogen, Carlsbad, CA, USA) following the manufacturer's protocol. Then, for the quality control, the RNA purity and concentration were measured using a Nano Drop 2000c spectrophotometer (Thermo Fisher Scientific, Waltham, MA). RNA samples were stored at -80 °C for further use. According to the protocol for the reverse transcription reaction, 3 μg total RNA was used as the template and the cDNA synthesis kit (Thermo Fisher Scientific, Waltham, MA) was used to synthesize its single strand cDNA. The reactions were performed with the following conditions: 5 min at 70 °C, 60 min at 42 °C, and 10 min at 70 °C. Then, these reaction tubes were moved onto ice for the qRT-PCR. Complying with the manufacturer's instructions, qRT-PCR was performed using the SYBR Green Supermix (Bio-Rad, Hercules, CA, USA) in the CFX96 Touch™ System (Bio-Rad). Primers used for qRT-PCR are listed in the Additional file 1: Table S1. The qRT-PCR was carried out according to the following protocol: 95 °C for 2.5 minutes, 40 cycles of 60 °C for 30 s, 5 s at 95 °C, 5 s at 65 °C, 5 s at 95 °C, and the melt curve (65–95 °C) with increments of 0.5 °C per cycle. All quantitative measurements were in triplicate and normalized to the internal glyceraldehyde-3-phosphatedehydrogenase (GAPDH). Data were analyzed using the $2^{-\Delta\Delta CT}$ method.

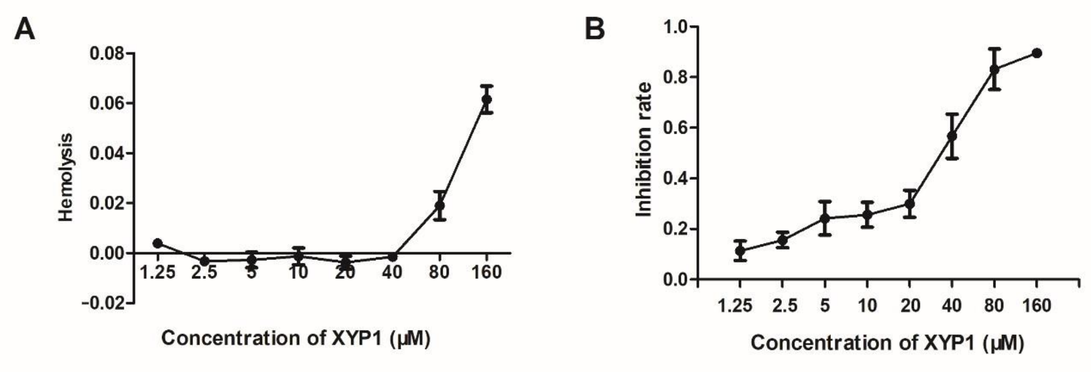

Figure S1. Hemolysis and cytotoxicity of XYP1. (**A**) Hemolysis of XYP1 on human blood red cells. 100 μL of XYP1 solution with two-fold serial dilutions (160 to 1.25 μM) was added to 100 μL RBCs in sterile 96-well plates, which were then incubated for 30 min at 37 °C and centrifuged at 1000 × g for 5 min. Aliquots (100 μL) of supernatant were transferred to sterile 96-well plates and hemoglobin release was measured using a Microplate Reader by absorbance at 540 nm. Data represent mean ± SE of three individual experiments. (**B**) Cytotoxicity of XYP1 determined by MTT assays. HFFs were cultured in 96-well plates in the absence or presence of XYP1 with two-fold serial dilutions (160 to 1.25 μM) for 24 h. The results represented the inhibition rate of HFFs compared with the control. The means were determined by values obtained from three independent experiments.

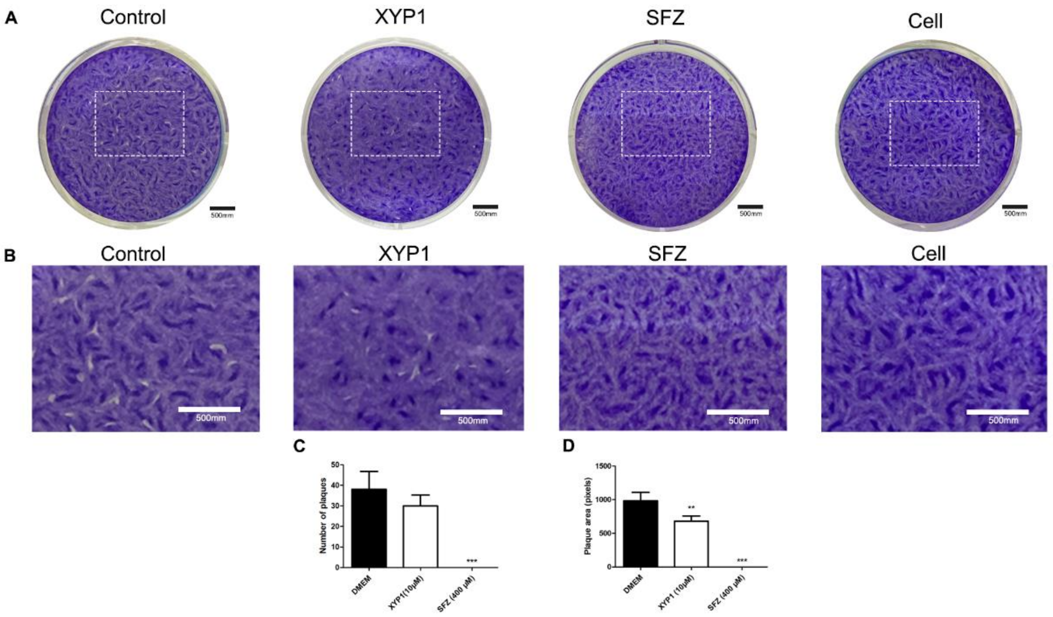

Figure S2. Plaque assay. HFFs were infected with tachyzoites and treated with DMEM (negative control), XYP1 (10 μM) and SFZ (positive control, 400 μM), respectively, for 7 days. (**A**) Photos of representative wells. (**B**) Magnified zones of plaques graphs. (**C**) The number of plaques determined manually. (**D**) Plaque area determined by Photoshop CS7. The means were determined by values obtained from three independent experiments (Student's two-tailed *t* test). *** $p < 0.01$ and *** $p < 0.001$ compared with the negative control. Scale bars = 500 mm.

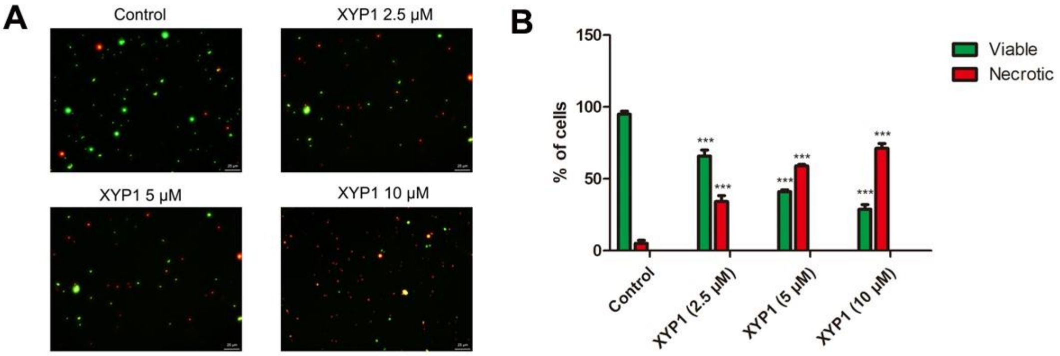

Figure S3. Fluorescent micrographs and quantification of AO/PI double-stained GFP-TgAtg8 strain tachyzoites. (**A**) Representative fluorescent micrographs. Untreated tachyzoites after 2 h showed large number of viable tachyzoites (green color) and very few apoptotic ones (yellow to orange color). XYP1-treated tachyzoites showed a large number of necrotic tachyzoites (bright red color) and few live tachyzoites. (**B**) Percentages of viable and necrotic tachyzoites after XYP1 treatment. Data presented as mean ± SEM from three separate experiments. *** $p < 0.0001$.

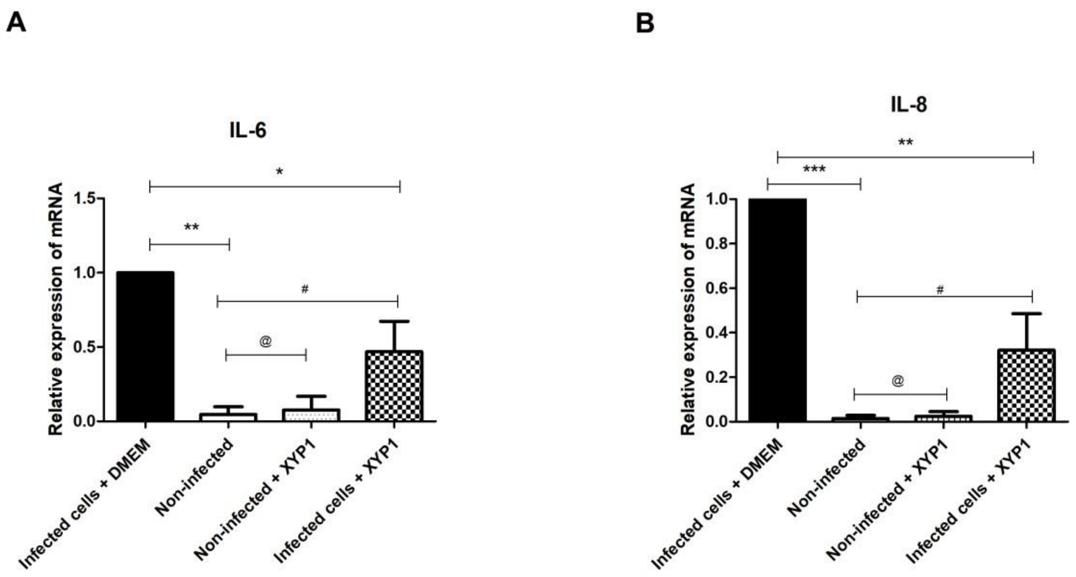

Figure S4. XYP1 affected the level of IL-6 and IL-8 in infected HFFs. (**A**) The mRNA level of IL-6. (**B**) The mRNA level of IL-8. HFFs with or without XYP1 treatment were considered as the non-infected and non-infected + XYP1, respectively. Host cells infected with parasites with DMEM- or XYP1-treatment were designated as infected cells + DMEM (negative control) and infected cells + XYP1, respectively. The means were determined by values obtained from three independent experiments (Student's two-tailed *t* test). $^{*}p < 0.05$, $^{**}p < 0.01$ and $^{***}p < 0.001$ compared with the negative control group. $^{\#}p < 0.05$ and $^{@}p > 0.05$ compared with the non-infected group.

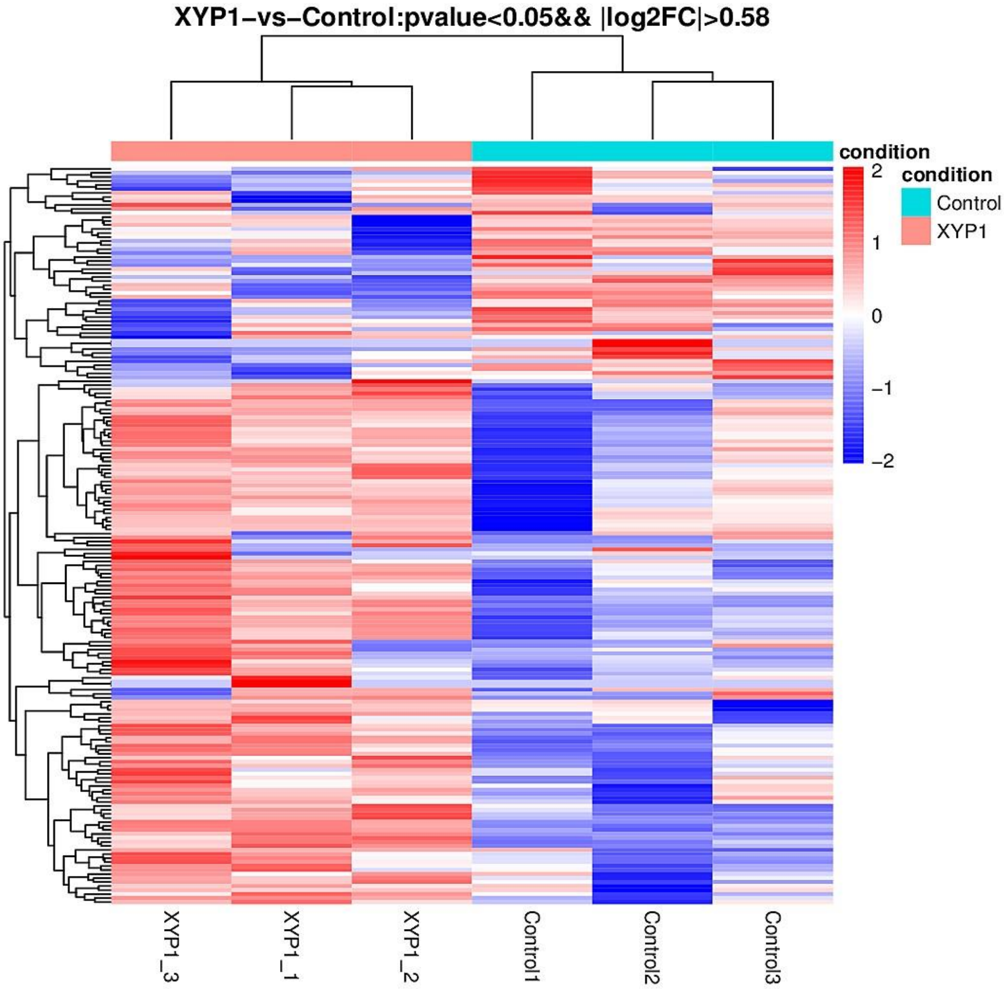

Figure S5. Hierarchical clustering of differentially expressed genes of *T. gondii* after 8 h XYP1 treatment. Expression values were $\log_2$-transformed and subsequently median-centered by gene. Rows were hierarchically clustered based on average linkage using Pearson correlation coefficients as the distance measure. The expression levels are visualized using gradient color scheme, the scale from least abundant to highest range is from −2.0 to 2.0. Green color indicates low expression, and red color indicates high expression of the detected genes. The left vertical axis represents sample ID. The horizontal axis shows clusters of samples and the above vertical axis shows clusters of DEGs.

Table S1. List of primers used in quantitative real-time PCR analysis.

GeneBank Accession	Primers	Sequences
XM_018779774.1	rpb-10-F	5′-TCATTTCGCCGACAAGTCCACAG-3′
	rpb-10-R	5′-TGACGCCTCCAGAAGTGACTCTC-3′
XM_002367312.2	MIC10-F	5′-AGCTCGCTCTGTCCTCCTAGAAAG-3′
	MIC10-R	5′-ATGTGTTCTGCTGAGTTCGCTGAG-3′
XM_018782086.1	HSP29-F	5′-GCACAACAGGTGGCTACTGGTATC-3′
	HSP29-R	5′-AGTAGGCGTCAAGGCGAGGTC-3′
AF265361.1	GAPDH (*T. gondii*)-F	5′-GCGAAGTCAGCCACAAGGA-3′
	GAPDH (*T. gondii*)-R	5′-CGGTGGACTCGCAGATGTAG-3′
NM_000600.5	IL-6-F	5′-GAAGAGCGCCGCTGAGAAT-3′
	IL-6-R	5′-GTGCAGAGGGTTTAATGTCAACT-3′
NM_001354840.3	IL-8-F	5′-TTTTGCCAAGGAGTGCTAAAGA-3′
	IL-8-R	5′-AACCCTCTGCACCCAGTTTTC-3′
NM_002046.7	GAPDH (Human)-F	5′-GGAGCGAGATCCCTCCAAAAT-3′
	GAPDH (Human)-R	5′-GGCTGTTGTCATACTTCTCATGG-3′
